# The “GPA race” as a public health issue: proactive resilience building in universities

**DOI:** 10.3389/fpubh.2026.1814686

**Published:** 2026-04-07

**Authors:** Yuhua Guo, Bo Li

**Affiliations:** College of Liberal Arts, Qufu Normal University, Qufu, China

**Keywords:** academic stress, health promoting universities, mental health prevention, psychological resilience, public health, university students

## 1 Introduction

The mental health of university students has become a pressing global public health concern. The transition to higher education coincides with a peak age of onset for many mental disorders, and the prevalence of anxiety, depression, and burnout among this population is alarmingly high (1, 2). Table 1 summarizes prevalence estimates from recent epidemiological studies.

**Table 1:** Prevalence of common mental health problems among university students.

Condition/Impact	Prevalence range	Population	References
Any mental disorder (12-month)	31.4%	First-year students in 8 countries	Auerbach et al. (1)
Any depressive or anxiety disorder	15.6%	U.S. undergraduates	Eisenberg et al. (2)
Severe role impairment among those with any disorder	42.9%	First-year students in 8 countries	Alonso et al. (17)

While multiple factors contribute to this crisis, a pervasive and intensifying driver is the extreme pressure surrounding academic achievement, often symbolized by the Grade Point Average (GPA)—a phenomenon increasingly referred to in educational discourse as the “GPA race” (3). This term, popularized in educational discourse to describe the intensifying competition for grades, reflects a broader phenomenon documented in the academic literature: the chronic stress associated with performance-based assessment cultures (4, 5) and the psychological risks of a fixed mindset centered on a singular metric of success (6). This “GPA race” has transformed a measure of learning into a chronic stressor that threatens the wellbeing of an entire generation of young people. We argue that this academic pressure cooker is not merely an educational problem but a significant public health issue requiring systemic, preventative action. This article posits that higher education institutions must pivot from a reactive, treatment-focused model to a proactive one that integrates psychological resilience building as a core component of their public health strategy, thereby safeguarding population health on campus.

## 2 The university as a critical setting for public health intervention

The university campus is a unique and influential setting for public health. It is a defined community where young adults spend a critical developmental period, and where health behaviors and trajectories can be established. The World Health Organization's Health Promoting Universities framework explicitly recognizes the role of higher education in fostering the health and wellbeing of students and staff (7). Within this framework, the mental health crisis among students is not just a concern for counseling centers but a core indicator of the health of the campus population and the institution's overall climate.

We posit that the “GPA race” functions as a potent, setting-specific stressor (4, 5). It operates as a chronic, low-to-moderate intensity stressor that erodes coping resources over time. The relentless focus on a singular metric of success can foster perfectionism, performance anxiety, and a fixed mindset, which are known risk factors for psychological distress (6). The differential impact of this stressor is evident across academic disciplines. The elevated rates of burnout and distress in highly competitive fields (Table 2) necessitate targeted interventions.

**Table 2:** Recent meta-analytic evidence on mental health by academic field.

Academic field	Key finding	References
Medicine	Global burnout prevalence: 35.8% (95% CI 25.7–45.8)	Jahrami et al. (18)
Medicine	Global burnout prevalence: 37.23% (95% CI 32.66–42.05)	Almutairi et al. (19)
Engineering	44.4% screened positive for depression/anxiety (*N* > 50,000)	Whitwer et al. (20)
Nursing	Burnout negatively impacts academic performance and wellbeing	Alsararatee et al. (21)
Law	Lawyer burnout: 42%−51% (industry survey)	Bloomberg Law (22)

When a significant proportion of the student population is experiencing clinically significant levels of stress, it constitutes a population-level health burden. This manifests not only in individual suffering but also in broader consequences such as academic attrition, reduced productivity, and increased demand on campus health services, highlighting the systemic nature of the problem.

## 3 Resilience: a malleable target for population-level prevention

Given the scale of the challenge, interventions targeting the entire student population—or large sub-groups—are more feasible and ethical than only treating those who meet diagnostic thresholds. Psychological resilience, defined as the capacity to adapt positively in the face of adversity, is an ideal target for such universal prevention (8). Crucially, contemporary research conceptualizes resilience not as a fixed personality trait, but as a dynamic, malleable process that can be learned and strengthened (9). This reframing opens the door for public health interventions designed to build protective factors across the entire campus community.

Key theoretical models support this approach. The Transactional Model of Stress and Coping emphasizes that an individual's cognitive appraisal of a stressor (e.g., viewing a low grade as a catastrophe vs. a learning opportunity) is central to their emotional and behavioral response (10). This appraisal process is modifiable through skills training, forming the theoretical foundation for cognitive-behavioral interventions that teach students to identify and reframe maladaptive thoughts about academic performance. Furthermore, the broaden-and-build theory of positive emotions suggests that cultivating positive emotions can broaden an individual's thought-action repertoire and build enduring personal resources, creating an upward spiral of wellbeing (11). These mechanisms—cognitive reframing and positive emotion cultivation—are teachable skills that can form the basis of a public health intervention.

## 4 A public health blueprint for resilience: moving beyond the clinic

To translate this theoretical understanding into practice, we propose a multi-level public health approach to resilience building on campus. This moves beyond individual therapy to create a supportive ecosystem.

First, at the universal prevention level, institutions can integrate resilience skills training into the core curriculum. This could take the form of for-credit courses or mandatory workshops for all 1^st^-year students. Notably, many universities already offer 1^st^-year orientation or wellness courses; embedding resilience training into these existing structures could enhance feasibility and scalability (12). These programs, drawing on evidence-based techniques from cognitive-behavioral therapy, mindfulness, and positive psychology, would teach students practical skills for managing stress, reframing challenges, regulating emotions, and building supportive relationships (13). This normalizes the concept of mental fitness, equipping all students with a psychological toolkit before distress escalates. Institutions such as the University of British Columbia (Wellness Peer Program) and Penn State (MINDSTRONG) have begun implementing such models, with Penn State's program providing early evidence of feasibility and acceptability: 75% of participants met program objectives and 91% reported encouraged interactions (14).

Second, at the selective prevention level, targeted programs can be offered to students identified as being at higher risk, such as those in highly competitive majors (see Table 2) or those showing early signs of academic burnout. These group-based interventions can provide a supportive space to practice skills and build peer connections, fostering a sense of belonging and shared experience. This approach is cost-effective and leverages social support as a key resilience resource, as emphasized by social-ecological models (15).

Third, a public health approach necessitates environmental-level interventions. Universities must critically examine the institutional drivers of the “GPA race.” This includes promoting assessment methods that value growth and learning over high-stakes, one-off exams; training faculty to recognize and respond to student distress with empathy; and fostering a campus culture that celebrates diverse forms of success and wellbeing. This shifts the focus from solely changing the individual to changing the environment that creates the stress. Faculty members play a critical role in shaping classroom culture and assessment practices (16). Without their engagement and support, resilience-building initiatives risk being superficial or ineffective. Therefore, faculty development programs should include training on recognizing student distress, fostering inclusive teaching practices, reducing unnecessary academic stressors, and designing assessments that promote learning rather than performance anxiety.

## 5 Discussion and implications for policy

Reframing the student mental health crisis as a public health issue with roots in the academic environment has profound implications. It calls for a shift in responsibility from overburdened counseling centers to a whole-of-university approach. We contend that investing in proactive, population-level resilience programs is not only an ethical imperative but also a strategic investment. By enhancing the coping capacity of the entire student body, universities can reduce the prevalence of severe distress, decrease demand for crisis services, improve academic outcomes and retention, and foster a healthier, more productive campus community.

The implementation of such a model faces challenges. It requires interdisciplinary collaboration between academic affairs, student services, and public health professionals. It demands funding and resources, and a cultural shift away from a purely remedial model of mental health. Critically, this cultural shift must include faculty, who are the primary architects of the academic environment and whose buy-in is essential for meaningful change. However, the cost of inaction—in terms of human suffering and lost potential—is far greater.

Future work should focus on implementation science to identify the most effective and sustainable ways to integrate resilience training into diverse university contexts. Research should also explore the long-term impact of these programs on population health metrics, academic trajectories, and post-graduation outcomes. Ultimately, by treating the “GPA race” as the public health challenge it is, and by proactively building resilience, higher education can fulfill its fundamental mission of fostering not just intellectual growth, but the holistic development of healthy, capable, and resilient individuals.
